# Strain Regulation and Defect Passivation of FA‐Based Perovskite Materials for Highly Efficient Solar Cells

**DOI:** 10.1002/advs.202305582

**Published:** 2023-12-08

**Authors:** Linfeng Zhang, Guohui Luo, Weihao Zhang, Yuxin Yao, Penghui Ren, Xiuhong Geng, Yi Zhang, Xiaoping Wu, Lingbo Xu, Ping Lin, Xuegong Yu, Peng Wang, Can Cui

**Affiliations:** ^1^ Key Laboratory of Optical Field Manipulation of Zhejiang Province Department of Physics Zhejiang Sci‐Tech University Hangzhou 310018 China; ^2^ State Key Laboratory of Silicon and Advanced Semiconductor Materials & School of Materials Science and Engineering Zhejiang University Hangzhou 310027 China

**Keywords:** defect passivation, ion migration, perovskite solar cells, stability, strain regulation

## Abstract

Formamidine lead triiodide (FAPbI_3_) perovskites have attracted increasing interest for photovoltaics attributed to the optimal bandgap, high thermal stability, and the record power conversion efficiency (PCE). However, the materials still face several key challenges, such as phase transition, lattice defects, and ion migration. Therefore, external ions (e.g., cesium ions (Cs^+^)) are usually introduced to promote the crystallization and enhance the phase stability. Nevertheless, the doping of Cs^+^ into the A‐site easily leads to lattice compressive strain and the formation of pinholes. Herein, trioctylphosphine oxide (TOPO) is introduced into the precursor to provide tensile strain outside the perovskite lattice through intermolecular forces. The special strain compensation strategy further improves the crystallization of perovskite and inhibits the ion migration. Moreover, the TOPO molecule significantly passivates grain boundaries and undercoordinated Pb^2+^ defects via the forming of P═O─Pb bond. As a result, the target solar cell devices with the synergistic effect of Cs^+^ and TOPO additives have achieved a significantly improved PCE of 22.71% and a high open‐circuit voltage of 1.16 V (voltage deficit of 0.36 V), with superior stability under light exposure, heat, or humidity conditions.

## Graphical abstract

High‐quality FA‐based perovskite films with few lattice defects, free of strain and suppressed ion migration are successfully obtained by adding Cs^+^ and TOPO molecule simultaneously, resulting in a high‐power conversion efficiency of 22.71% for solar cells with a significantly improved operational stability.

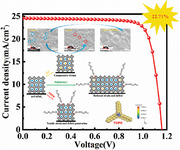

## 1 Introduction

Over the past decade, the power conversion efficiency (PCE) of single‐junction perovskite solar cells (PSCs) has undergone an amazing growth from 3.8%^[^ ^1^ ^]^to 26.1%.^[^ ^2^ ^]^ The remarkable achievements are closely related to the unique optoelectronic properties of perovskite materials, including the tunable bandgap, low exciton binding energy, and high carrier mobility.^[^ ^3^ ^]^ The structural formula of perovskite is ABX_3_, where A represents a monovalent cation, e.g., methylammonium (MA^+^), formamidine (FA^+^), or cesium ion (Cs^+^); B denotes the bivalent metal cation (Pb^2+^ or Sn^2+^); X stands for the halide anion (I^−^, Br^−^, or Cl^−^). Currently, the PSCs with PCEs exceeding 25% are mostly achieved based on FAPbI_3_ material,^[^ ^4^ ^]^ owing to its suitable bandgap, low carrier recombination, and high thermal stability.^[^ ^5^ ^]^

However, the large‐size FA^+^ easily causes the instability of crystal structure, leading to a significant increase in lattice defects and ion migration in perovskite materials.^[^ ^6^ ^]^ Besides, the pure phase α‐FAPbI_3_ prefers to transform into the non‐photoactive δ phase at room temperature.^[^ ^7^ ^]^ The lattice structure disorder or phase instability of FAPbI_3_ is aggravated by the surrounding environmental stimulus (e.g., light, heat, or humidity). In order to promote the perovskite crystallization and stabilize the lattice, external ions such as MA^+^, Cs^+^, methylenediammonium ions (MDA^2+^), Br,^−^ and Cl^−^ are usually introduced into the FA^+^‐based perovskite structure. Among them, MACl is generally used as an additive to facilitate the crystallization and grain enlargement of FAPbI_3_ perovskite.^[^ ^8^ ^]^ Nevertheless, most of MACl tends to volatilize during the annealing stage for the film formation, and only a small amount of MA^+^ is left at the A‐site, leading to limited regulation of lattice strain and even increased vacancies defects due to the volatilization of MA^+^ at elevated temperature.^[^ ^9^ ^]^

Cs^+^ is another choice for boosting the crystallization and stabilizing the lattice of FAPbl_3_ perovskite. In particular, the introduction of CsI in the two‐step sequential deposition method effectively inhibits the generation of residual PbI_2_ and δ‐FAPbI_3_, resulting in the enhancement of perovskite film quality and phase stability.^[^ ^10^ ^]^ However, the Cs^+^ with a smaller size than FA^+^ induces compressive strain in the lattice, which could hinder the further improvement in device performance. Moreover, the reaction between CsI and PbI_2_ leads to the generation of δ‐CsPbI_3_ phase with a sparse structure, resulting in the formation of uneven pinholes in the film. Previous studies have introduced larger size MDA^2+^ or guanidinium ions (GA^+^) and Cs^+^ simultaneously to FAPbI_3_ lattice to regulate the crystallization kinetics, thereby reducing the lattice strain and the appearance of pinholes in the film.^[^ ^11^ ^]^ However, few organic cations can be adopted to replace the FA^+^ ions due to the limitation of the tolerance factor.^[^ ^12^ ^]^ Moreover, the co‐doping ions to occupy the A‐site with nonuniform distribution at a microscopic scale can initiate the aggregation of cations and decrease the device performance.^[^ ^13^ ^]^

In this work, we successfully prepared high‐quality FA‐based perovskite films free of lattice strain by combining A‐site doping of Cs^+^ and the out‐of‐lattice tensile effect of a large trioctylphosphine oxide (TOPO) molecule. Moreover, the P═O functional group and long alkyl chain structure of TOPO passivate defects within the perovskite films and promote the phase stability. Based on the novel strategy of strain modulation and defect passivation, a significantly improved PCE of 22.71% has been achieved for PSC devices with a low open‐circuit voltage (*V*_OC_) deficit of 0.36 V.

## 2 Results and Discussion

**Figure 1:**
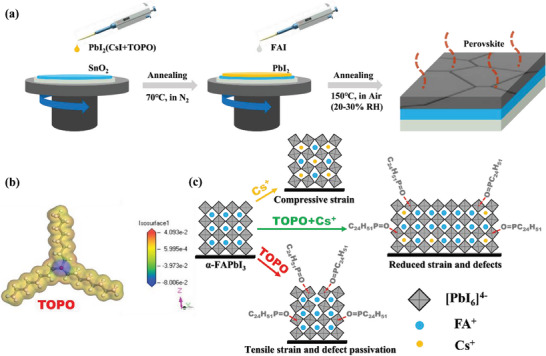
a) Schematic diagram of preparing perovskite films using the two‐step sequential method. b) ESP map of trioctylphosphine oxide (TOPO). The blue and the red indicate electronegative and electropositive parts, respectively. c) The mechanism diagram of lattice strain regulation and/or defect passivation added with CsI and/or TOPO.

The schematic preparing process of FA‐based perovskite films is shown in **Figure** **1**a. First, the PbI_2_ precursor added with appropriate concentrations of CsI and TOPO was spin‐coated onto the substrate, and annealed at 70 °C for 1 min in nitrogen (N_2_) atmosphere. Afterward, organic amine salt solutions of FAI with 25 mol% MACl dissolved in isopropyl alcohol (IPA) were spin‐coated onto the PbI_2_ layer, and then annealed in an air atmosphere (≈30% relative humidity (RH)) to form the perovskite films. To explore the intrinsic charge distribution of TOPO molecule, the electrostatic potential (ESP) was calculated by the first‐principles density functional theory (DFT). Figure 1b illustrates that the color of the ESP map changes from yellow of the alkyl chain to blue of P═O functional group, which indicates that the electron charges of the molecule are highly concentrated on the ─P═O group, conducive to the strong interaction with Pb^2+^ of perovskite precursor.^[^ ^14^ ^]^ As shown in Figure S1 (Supporting Information), The interaction energy ($E_{int}$) of TOPO and PbI_2_ obtained with DFT calculation is as high as 17.68 Kcal mol^−1^, which is higher than that of TOP and PbI_2_ (17.05 Kcal mol^−1^), indicating that more effective passivation of defects and relaxation of the strain in the perovskite by the former. Figure 1c illustrates the interaction mechanism of Cs^+^ and TOPO additives with the FAPbI_3_ lattice. The Cs^+^ has an effective radius of 167 pm, which is smaller than that of the FA^+^ (253 pm).^[^ ^15^ ^]^ Therefore, after doping a certain amount of CsI in the α‐FAPbI_3_ crystal, the local lattice generates a compressive strain. On the other hand, the TOPO molecule not only suppresses defects due to the strong interaction of the ─P═O group with undercoordinated Pb^2+^, but induces a tensile strain outside the lattice through intermolecular forces. We thus expect that high‐quality perovskite films with a reduction of lattice defects and strain can be obtained via the strain‐compensation regulation method of the incorporation of Cs^+^ and TOPO simultaneously.

**Figure 2:**
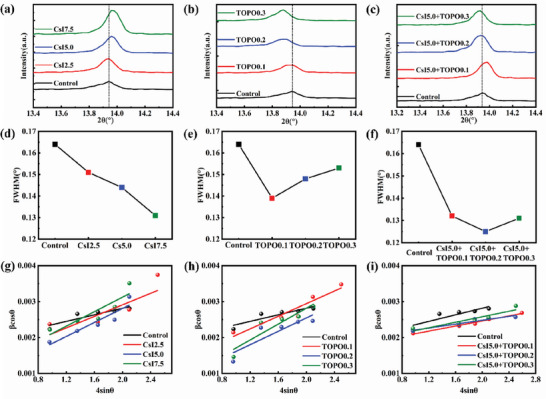
XRD patterns of perovskite films: a) control and Cs^+^ doped (2.5 mol% CsI (CsI2.5), 5.0 mol% CsI (CsI5.0), and 7.5 mol% CsI (CsI7.5)); b) added with TOPO (0.1 mol% TOPO (TOPO0.1), 0.2 mol% TOPO (TOPO0.2) and 0.3 mol% TOPO (TOPO0.3)) and c) added with CsI5.0 and TOPO with different concentrations. d–f) The FWHM for the corresponding perovskite films. g–i) The W–H plots of the corresponding perovskite films.

The X‐ray diffraction (XRD) measurement of the perovskite films added with varing concentrations of Cs^+^ was conducted (**Figure** **2**a; Figure S2, Supporting Information). In contrast to the control film, the 2*θ* of the (100) plane of the cubic perovskite phase gradually shifts to a larger angle with the increase of Cs^+^ content. It indicates that lattice compression strain increases due to a smaller size of Cs^+^ doped into the A‐site, according to the Bragg diffraction equation, 2*d*sin*θ* = *nλ*, where *λ* is the wavelength of the X‐ray, *d* is the crystal plane spacing and *θ* is the incident angle.^[^ ^11a,b^ ^]^ Interestingly, unlike the incorporation into the perovskite lattice of Cs^+^, the TOPO still achieves a stretching effect outside of the lattice through intermolecular forces, which can be seen in Figure 2b. As the content of TOPO increases, the (100) plane characteristic peak of films gradually shifts to a smaller diffraction angle. Note that the Cs^+^ doping obviously improves the performance of PSCs, and CsI5.0 sample (the molar ratio of CsI to PbI_2_ is 5%) has the most striking effect (Figure S3, Supporting Information). The TOPO with different concentrations are further added into the CsI5.0 sample, thereby the 2*θ* of (100) plane characteristic peak gradually decreases as the TOPO additive increases, and it becomes nearly the same as that of the control when the TOPO content is 0.2 mol% (CsI5.0+TOPO0.2), as shown in Figure 2c. It is thus reasonable to deduce that the perovskite layer is almost free of strain due to the compensation effect.

The variation of perovskite lattice strain can be estimated by using Williamson–Hall (W–H) equation, $\beta_{T}\cos\theta =\varepsilon \left(4\sin\theta \right)+\frac{k\lambda }{D}$, where *D* is the crystallite size, *k* is the Scherrer constant of 0.89, and ε is the lattice strain. The β_T_ is equal to the value of full width at half maximum (FWHM). The FWHM of the characteristic peaks of (100) plane for the corresponding films were estimated, as shown in Figure 2d–f. Generally, the FWHM reflects the crystallinity of the film, i. e., the smaller FWHM indicates the higher crystallinity. Compared with the control, either Cs^+^ or TOPO additives benefit for improving the crystallinity of perovskite films (Figure 2d,e). Moreover, the introduction of both additives with the appropriate concentrations (CsI5.0+TOPO0.2) has the most remarkable improvement effect (Figure 2f). The *ε* of the films are thus obtained by a linear fitting using the W–H formula.^[^ ^8^, ^11^ ^]^ By comparing the slopes of the linear fits, we can quantitatively analyze the perovskite lattice strain (Figure 2g–i; Figure S4, Supporting Information). The introduction of Cs^+^ or TOPO alone increases the lattice strain of the films (Figure 2g,h). Fortunately, the introduction of both additives significantly reduces the lattice strain, which achieves the smallest for the sample of CsI5.0+TOPO0.2 (Figure 2i; Figure S4, Supporting Information). The calculated parameters in detail are summarized in Table S1 (Supporting Information). Therefore, the results confirm that the synergistic effects of CsI and TOPO are quite effective to modulate the perovskite lattice strain with improved crystallization.

**Figure 3:**
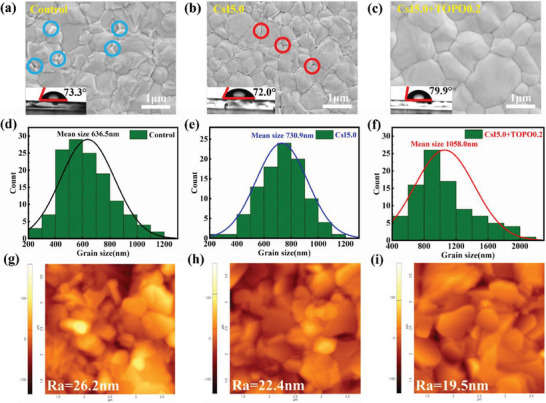
Top‐view SEM images of perovskite films: a) the control, b) CsI5.0, c) CsI5.0+TOPO0.2. Insets show the water contact angles for the corresponding film surfaces. The grain size statistics of perovskite films: d) the control, e) CsI5.0, f) CsI5.0+TOPO0.2. AFM images of perovskite films: g) the control, h) CsI5.0, i) CsI5.0+TOPO0.2.

To demonstrate the effect of Cs^+^ or TOPO on the crystallization of perovskites, top‐view and cross‐sectional scanning electron microscopies (SEM) of the films (control, CsI5.0 and CsI5.0+TOPO0.2) were observed, as shown in **Figure** **3**a–c and Figure S5 (Supporting Information). The surface of the control is uneven associated with relatively small grains and the mean grain size is 636.5 nm (Figure 3a,d). Besides, there are many residual PbI_2_ (white flakes marked by blue circles in Figure 3a) that did not completely react with FAI.^[^ ^16^ ^]^ The introduction of 5.0 mol% CsI is beneficial to improve the crystal quality with the increased grain size (mean grain size of 730.9 nm, Figure 3e), and moreover, the residues of PbI_2_ are effectively eliminated. The small amount of CsI additive can react with excess PbI_2_ to form δ‐CsPbI_3_, which serve as the nucleation centers to promote the crystallization of FAPbI_3_ films. However, the sparse structure of δ‐CsPbI_3_ is easy to cause the formation of pinholes at the grain boundaries of the perovskite films, shown in the regions with red circles (Figure 3b). The further introduction of TOPO into PbI_2_ precursor containing CsI effectively inhibits the generation of δ‐CsPbI_3_, due to the strong interaction of the P═O bond with Pb^2+^. Moreover, a dense perovskite film with a significant increase in grain sizes (the mean size of 1058.0 nm) is obtained, as shown in Figure 3c,f. Additionally, the water contact angle of the film added with TOPO molecule containing long alkyl chain is obviously larger than the others (insets in Figure 3a–c), improving the water resistance in the humidity condition.

The surface atomic force microscopy (AFM) of the corresponding perovskite films were also measured (Figure 3g–i). The averaged roughness (*R*_a_) of the control is 26.2 nm, and it reduces to 22.4 and 19.5 nm for the CsI5.0 and CsI5.0+TOPO0.2 samples, respectively. Normally, the smaller the surface roughness of the film, the closer the contact between the perovskite and carrier transport layers. The *R*_a_ value of the target film is smaller than the others, beneficial for carrier extraction or transport across the heterojunction contact.

**Figure 4:**
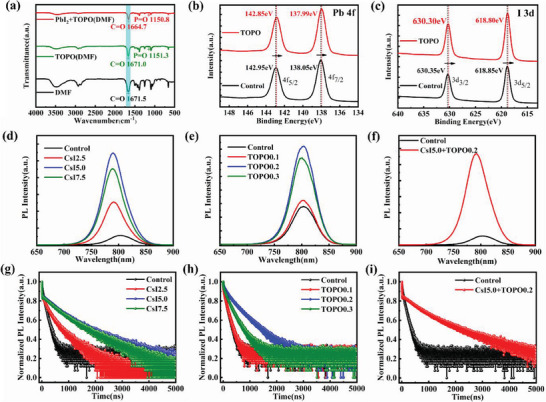
a) FTIR spectra for DMF, TOPO dissolved in DMF and PbI_2_+TOPO dissolved in DMF. The XPS spectra for the control and TOPO0.2 perovskite films: b) Pb 4f, c) I 3d. The PL spectra of perovskite films on glass substrates for d) the control and doped with CsI (CsI2.5, CsI5.0 and CsI7.5), e) added with TOPO (TOPO0.1, TOPO0.2 and TOPO0.3) and f) the target (CsI5.0+TOPO0.2). g–i) TRPL spectra of the corresponding perovskite films on glass substrates.

Fourier transform infrared (FTIR) and X‐ray photoelectron spectroscopy (XPS) measurements were performed to further investigate the interaction between the TOPO molecule and PbI_2_. **Figure** **4**a shows the FTIR spectra of TOPO and PbI_2_+TOPO in DMF solvent. The DMF solvent has a characteristic peak of C═O located at 1671.5 cm^−1^, which is almost unaffected with the introduction of TOPO (1671.0 cm^−1^). Besides, another characteristic peak of P═O located at 1151.3 cm^−1^ is observed. The further introduction of PbI_2_ leads to both the characteristic peaks of C═O and P═O moving toward lower wavenumber of 1664.7 and 1150.8 cm^−1^, respectively. It indicates that either C═O of DMF solvent or P═O of the TOPO additive has a strong coordination effect with the Pb^2+^. Figure 4b,c shows the XPS spectra of Pb 4f and I 3d for the perovskite films without or with TOPO molecule, respectively. The complete XPS spectra are shown in Figure S6 (Supporting Information). The main peak positions of Pb^2+^ in the control film are 142.95 and 138.05 eV, which move toward lower binding energies of 142.85 and 137.99 eV with the adding of TOPO molecule, respectively. Similarly, the corresponding characteristic peaks of I 3d spectra also shift from 630.35 and 618.85 eV to 630.30 and 618.80 eV, respectively. The shifts in the binding energies of Pb 4f and I 3d confirm the strong interaction between the TOPO and perovskite.^[^ ^17^ ^]^

The carrier recombination and dynamic behaviors of the films added with CsI or TOPO were analyzed by steady‐state photoluminescence (PL) and time‐resolved PL (TRPL) spectroscopies (Figure 4d–i). The stronger the PL intensity suggests that less non‐radiative recombination and fewer defects in the bulk perovskite. In contrast to the control, the PL intensities of the films containing different concentration of CsI increase, for which the CsI5.0 sample has the strongest signal (Figure 4d). Besides, the PL peaks of the films added with CsI generate obvious blue shifts, indicating that the bandgap increases due to the doping of Cs^+^ into the A‐site, which agrees with the absorption spectra (Figure S7, Supporting Information). The adding of TOPO molecule also enhances the PL intensities, and the sample TOPO0.2 has the most notable effect (Figure 4e). Note that the luminescence peaks of the films added with the TOPO shift blue slightly, which should be ascribed to its passivation effect. As expected, the PL intensity of the target film (CsI5.0+TOPO0.2) increases significantly (Figure 4f), indicating that the non‐radiative recombination is largely suppressed by the introduction of CsI and TOPO simultaneously. For the TRPL measurement, the PL intensity decay curve is fitted with the double‐exponential decay equation, *y* = *y*_0_+*A*_1_exp(‐*t*/*τ*_1_)+*A*_2_exp(‐*t*/*τ*_2_), where *y*_0_ is the curve offset, *A*_1_ and *A*_2_ are the amplitude; *τ*_1_ and *τ*_2_ represent the fast and slow decay components, associated with the trap‐assisted non‐radiative and radiative recombination processes, respectively.^[^ ^11c^ ^]^ The average carrier lifetime (*τ*_ave_) is calculated from the relation of *τ*_ave_ = (*A*_1_ *τ*_1_^2^+*A*_2_ *τ*_2_^2^)/(*A*_1_ *τ*_1_+*A*_2_ *τ*_2_). The fitted or calculated results in detail are summarized in Table S2 (Supporting Information). The *τ*_ave_ for the control perovskite film is 873.17 ns, and it increases to 3093.11 and 2108.17 ns for the CsI5.0 and TOPO0.2 samples, respectively (Figure 4g,h). Moreover, it further increases to 3214.33 ns with the introduction of CsI and TOPO simultaneously (Figure 4i). The significant improvement in PL intensity or *τ*_ave_ can be dominantly attributed to the enhanced crystallization and passivation of trap‐state defects, associated with the release of lattice strain. In addition, we explored the introduction of TOPO at the perovskite surface that passivates the defects and promotes the carrier transport simultaneously (Figures S8 and S9, Tables S3 and S4, Supporting Information), in agreement with the previous studies.^[^ ^18^ ^]^

**Figure 5:**
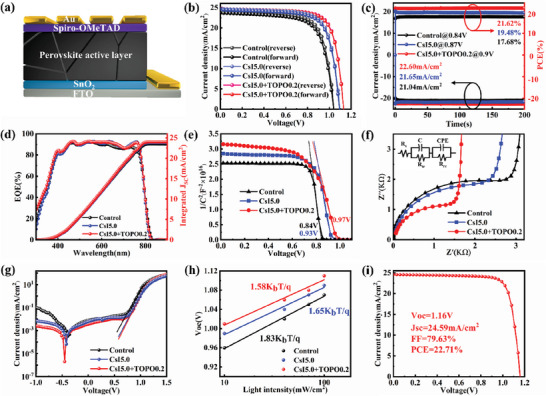
a) The *n*–*i*–*p* structure of PSC device. b) Illuminated *J–V* curves of the control, CsI5.0 and CsI5.0+TOPO0.2 devices with reverse and forward scans. c) Steady‐state current density and PCE output for the corresponding devices measured at the maximum power point. d) EQE and integrated *J*_SC_ of the corresponding devices. e) The Mott–Schottky measurement at 1m Hz for the corresponding devices. f) Nyquist plots of electrochemical impedance spectroscopy (EIS) under dark condition and g) The *J–V* characteristic curves of the control, CsI5.0 and CsI5.0+TOPO0.2 devices in dark. h) The *V*_OC_ dependence of light intensity for the corresponding devices. i) Illuminated *J–V* curves of the champion device prepared with the target film.

The PSCs with the regular *n*–*i*–*p* structure of FTO/SnO_2_/perovskite/Spiro‐OMeTAD/Au were prepared (**Figure** **5**a). Figure 5b shows the typical *J–V* curves of the control, CsI5.0 and the target (CsI5.0+TOPO0.2) devices under both the forward and reverse scans, with the output parameters listed in **Table** **1**. With the Cs^+^ doping, the *V*_OC_ is improved from 1.05 to 1.11 V, short‐circuit current density (*J*_SC_) is slightly increased from 24.11 to 24.37 mA cm^−2^, and fill factor (FF) is enhanced from 70.36% to 73.77%, resulting in an increase of PCE from 17.81% to 19.73%. Moreover, the target device has the *V*_OC_ of 1.17 V, *J*_SC_ of 24.55 mA cm^−2^ and FF of 76.01%, delivering a higher PCE of 21.71%. The statistical PCEs of the corresponding devices also exhibt a good repeatability (Figure S10, Supporting Information). The significant improvements of the PSC device performance (*V*_OC_, *J*_SC_, FF) are mainly ascribed to the suppressed carrier recombination and promoted carrier extraction/collection, originating from the enhanced crystallization, effective passivation of defects, and release of lattice strain by the synergistic effect of CsI and TOPO. In addition, the hysteresis of the devices is negligible with the H‐index of 0.032, 0.023 and 0.004 for the control, CsI5.0 and target (CsI5.0+TOPO0.2) devices, respectively. The photocurrent density and steady‐state PCE output of the corresponding devices at the maximum power point were measured (Figure 5c). The stable PCEs of the three different devices are 17.68%, 19.48% and 21.62%, with the photocurrent density of 21.04, 21.65, and 22.60 mA cm^−2^_,_ respectively. Note that the devices have a fast saturation response of PCE or current density, consistent with the small H‐index results. Figure 5d shows the external quantum efficiency (EQE) spectra of the three different devices. The integrated *J*_SC_ for the control, CsI5.0 and the target devices are 23.52, 23.85 and 24.06 mA cm^−2^, respectively, in consistence with the *J*_SC_ obtained from the illuminated *J–V* curves. Based on the fitted bandgap of the target FA‐based perovskite (1.52 eV) from the second derivative of EQE with respect to wavelength *λ* (Figure S11, Supporting Information),^[^ ^19^ ^]^ the *V*_OC_ deficit of the device is estimated to be 0.36 V.

**Table 1:** Photovoltaic parameters and hysteresis factor (H‐index) of the control, CsI5.0 and target (CsI5.0+TOPO0.2) devices.

		*J*_SC_ [mA cm^−2^]	*V*_OC_ [V]	FF [%]	PCE [%]	H‐index
Control	Reverse	24.11	1.05	70.36	17.81	0.032
	Forward	23.65	1.04	70.11	17.24	
CsI5.0	Reverse	24.09	1.11	73.77	19.73	0.023
	Forward	24.37	1.09	72.58	19.28	
target	Reverse	24.43	1.17	76.01	21.71	0.004
	Forward	24.55	1.17	75.22	21.62	

The built‐in voltage (*V*_bi_) of the devices is obtained by capacitance‐voltage (*C–V*) measurement (Figure 5e). The fitted *V*_bi_ of the target device (0.97 V) is significantly and slightly higher than that of the control (0.84 V) and the CsI5.0 device (0.93 V), respectively. The Mott–Schottky (M–S) measurement with the greater *V*_bi_ of the device indicates that the higher quality of the perovskite film.^[^ ^20^ ^]^ The electrochemical impedance spectra (EIS) of the devices are shown in Figure 5f. The target device has the smallest series resistance (*R*_s_) and the largest recombination resistance (*R*_rec_), which are predominately attributed to the improvement of perovskite film quality with enhancement of charge carrier extraction and suppression of non‐radiative recombination.^[^ ^21^ ^]^ In addition, the promotion of hole extraction or transport can be ascribed to a better energy band alignment obtained for the target perovskite (Figure S12, Supporting Information). Figure 5g shows the *J–V* curves of the PSC devices in dark. It can be seen that the *J*_0_ of the target device is lower than the two others. The *V*_OC_ of the corresponding devices as a function of the light intensity is shown in Figure 5h. The obtained ideal factor *n* values of the control, CsI5.0 and CsI5.0+TOPO0.2 devices are 1.83, 1.65 and 1.58, respectively. Both the reduction of *J*_0_ and *n* indicate that the non‐radiative recombination is suppressed, and thus the *V*_OC_ loss is reduced. Based on the improved growth of FA‐based perovskite films with less defects, free of strain and the optimized device processing, a champion PCE of 22.71% for the target device has been achieved (*V*_OC_ of 1.16 V, *J*_SC_ of 24.59 mA cm^−2^ and FF of 79.63%), as shown in Figure 5i.

**Figure 6:**
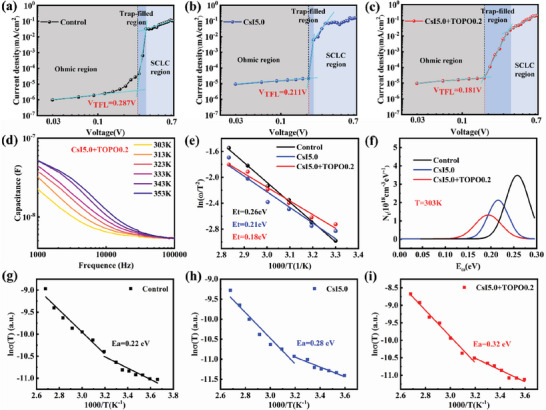
SCLC measurements of electron‐only devices based on the perovskite films: a) the control, b) CsI5.0, and c) CsI5.0+TOPO0.2. The device structure is ITO/SnO_2_/perovskite/PCBM/Ag. d) Capacitance–frequency curves of the target device (CsI5.0+TOPO0.2) at varing temperatures. e) Arrhenius plots of the characteristic transition frequencies of the devices. f) The fitted tDOS distribution of the devices measured at 303 K. Temperature‐dependent conductivity σ(T) of the films: g) the control, h) CsI5.0 and i) CsI5.0+TOPO0.2.

To further explore the defect reductions for the suppressed carrier recombination behaviors, the space charge limited current (SCLC) technique and thermal admittance spectroscopy (TAS) were applied for the three different perovskite films. The trap‐state defect density (*N*_trap_) is obtained by using the perovskite single‐electron devices with the structure of ITO/SnO_2_/perovskite/PCBM/Ag, based on the following formula:

(1) $$V_{\mathrm{TFL}}=\frac{L^{2}eN_{\mathrm{trap}}}{2\varepsilon \varepsilon_{0}}$$

where *V*_TFL_ is the fitted trap filled limit voltage, *L* is the thickness of perovskite films, *e* is the elementary charge, *ε* is the relative dielectric constant of perovskite and *ε*_0_ is the vacuum permittivity. The *V*_TFL_ of the control, CsI5.0 and CsI5.0+TOPO0.2 films are 0.287, 0.211 and 0.181 V, respectively (**Figure** **6**a–c). The calculated *N*_trap_ of the corresponding films are 4.44 × 10^15^, 3.27 × 10^15^, and 2.80 × 10^15^ cm^−3^, respectively. Moreover, the trap density of state (tDOS) and the defect level position of perovskite films are further analyzed using the TAS,^[^ ^22^ ^]^ as shown in Figure 6d–f. First, the capacitance‐frequency spectra of the different devices in the temperature range of 303–353 K were measured (Figure 6d; Figure S13, Supporting Information). Then, the defect trap energy level (*E*_t_) is derived from the following formula,

(2) $$\omega_{0}=\beta T^{2}\exp\left(-\frac{E_{t}}{K_{b}T}\right)$$

where ω_0_ is the characteristic transition frequency from the peak value of the [‐ ω × dC/dω] curve obtained from Figure 6d and Figure S9 (Supporting Information), β is temperature independent parameter, *K*_b_ is the Boltzmann constant, and *T* is the temperature. The trap density *N*_t_ can be obtained by the equation:

(3) $$N_{t}=-\frac{V_{bi}}{eW}\;\frac{dC}{d\omega }\frac{\omega }{K_{b}T}$$

where *C* is the capacitance, *ω* is the applied frequency and *W* is the depletion region width. The *V*_bi_ and *W* are obtained from the *C‐V* measurement (Figure 5e). Figure 6e shows that the fitted *E*_t_ of the control, CsI5.0, and the target devices are 0.26, 0.21, and 0.18 eV, respectively. Moreover, the energetic trap density distribution peaks of the control, CsI5.0 and target devices at the temperature of 303 K are 0.26 × 10^18^, 0.21 × 10^18^, 0.18 × 10^18^ cm^−3^ eV^−1^, respectively (Figure 6f). The detected defects using the TAS analysis are presumably *I*_Pb_ and *Pb*_I_ anti‐site defects with the *E*_t_ ranging between 0.1 and 0.3 eV.^[^ ^23^ ^]^ The introduction of Cs^+^ and TOPO significantly suppresses or passivates the trap defects with the reduced *E*_t_ and *N*_t_. Therefore, the carrier recombination of perovskite films is effectively suppressed and the film quality is largely improved, which agree well with the PL, TRPL and SCLC results mentioned above.

Besides, ion migration originated from the mobile I^−^ in the perovskite films could limit the device performance and operational stability.^[^ ^24^ ^]^ By measuring the conductivity of perovskite films at different temperatures, the ion migration activation energy (*E*_a_) is extracted by the following Arrhenius equation:

(4) $$\sigma =\frac{\sigma_{0}}{T}\exp\times \left(\frac{-E_{a}}{K_{b}T}\right)$$

where *σ* is the conductivity at the given absolute temperature *T*, and *σ*_0_ is the pre‐exponential factor. The temperature‐dependent conductivity measurements with the architecture of Ag/perovskite/Ag were performed (Figure 6g–i). The obtained *E*_a_ for the control, CsI5.0 and CsI5.0+TOPO0.2 films are 0.22, 0.28 and 0.32 eV, respectively. The *E*_a_ of the target film is obviously increased, which indicates that the ion migration is greatly impeded due to the release of lattice strain. Note that the *E*_a_ of the CsI5.0 film increases while it decreases for the TOPO0.2 sample (0.19 eV, Figure S14, Supporting Information), in contrast to the control. The ion migration is hindered by the Cs^+^ doping due to the introduced compressive strain, whereas it is promoted by the tensile strain by the added TOPO, in agreement with the previous studies.^[^ ^25^ ^]^

**Figure 7:**
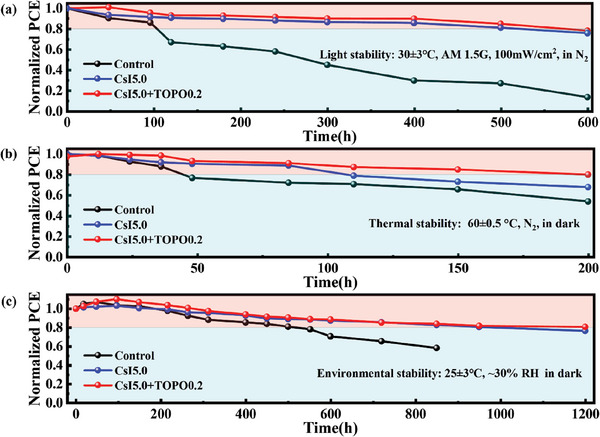
a) Light stability of the unencapsulated control, CsI5.0 and CsI5.0+TOPO0.2 PSCs measured under one‐sun illumination (AM 1.5G, 100 mW cm^−2^, in N_2_, 30 ± 3 °C). b) Thermal stability of the unencapsulated control, CsI5.0 and CsI5.0+TOPO0.2 PSCs heated at 60 °C in N_2_ environment. c) The storage stability of the corresponding PSCs exposed in ambient air (25 ± 3 °C, relative humidity (RH) of ≈30%) and in dark.

The stability of PSCs devices is another important issue. To investigate the operational stability of the PSCs (the control, CsI5.0 and the target (CsI5.0+TOPO0.2)), they were immersed under simulated one‐sun irradiation (AM 1.5G, 100 mW cm^−2^, 30 ± 3 °C) in N_2_ atmosphere for 600 h (**Figure** **7**a). The *T*_80_ lifetime of the control, CsI5.0 and target devices are 104, 522 and 576 h, respectively. The improved light stability of unencapsulated target devices can be attributed to the remarkable strain release to inhibit ion migration and perovskite decomposition. Figure 7b shows the normalized PCEs of the unencapsulated PSCs for thermal stability measurement. After heating at 60 °C on a hotplate for 200 h in N_2_ atmosphere, the target device maintains 80.0% of its initial PCE. In contrast, the control and CsI5.0 devices exhibit faster decline in PCEs to 53.9% and 67.8%, respectively. The doping of Cs^+^ improves the thermal stability of the device, consistent with previous reports.^[^ ^26^ ^]^ The introduction of the TOPO additive further improves the thermal stability, which should be closely related to the enhancement of the structural stability of the perovskite and the release of lattice strain, leading to the suppression of the thermal activation degradation of perovskite films. The environmental stability of PSCs was also investigated. The unencapsulated PSCs were stored in ambient air and in dark at a temperature of 25 ± 3 °C and the RH of ≈30% (Figure 7c). The *T*_80_ lifetime of the control, CsI5.0 and the target devices are 500, 950 and 1200 h, respectively. The environmental stability improvement of the devices should be attributed to the enhancement of crystallization by the Cs^+^ doping, which is further promoted owing to the protection of long carbon chain of TOPO and the stabilization of lattice.

## 3 Conclusion

In summary, high‐quality FA‐based perovskite films with few lattice defects and free of strain are successfully obtained by adding Cs^+^ and TOPO molecule simultaneously. The doping of Cs^+^ into A‐site promotes the crystallization and phase stability of α‐FAPbI_3_ films, whereas pinholes and local compressive strain inevitably generate. The introduction of TOPO molecule containing long alkyl chain and P═O bond not only induces tensile strain in the lattice, but passivates the undercoordinated Pb^2+^ defects. The synergistic effects of Cs^+^ and TOPO molecule with the optimized concentration effectively eliminate the strain and suppress the non‐radiative recombination originated from the lattice defects (*I*_Pb_ and *Pb*_I_). Based on the special strategy of strain modulation and defect passivation, a significantly improved PCE of 22.71% has been achieved for PSC devices with a small *V*_OC_ deficit of 0.36 V. Moreover, the long‐term stabilities of unencapsulated devices are obviously improved, which retain above 80% of their initial PCE values after exposing to simulated one‐sun illumination in N_2_ atmosphere for 600 h, heating at 60 °C in N_2_ atmosphere for 200 h, and storing in air with a RH of ≈30% for 1200 h, respectively. Our work provides a feasible strategy for modulating lattice strain and reducing defects in FA‐based perovskite for high performance photovoltaic applications.

## 4 Experimental Section

### Materials

Fluorine‐tin‐oxide (FTO) glass substrates (7 Ω sq^−1^) were purchased from Advanced Election Technology Co., Ltd. Tin (IV) oxide colloid (15% colloidal dispersion in water) was purchased from Alfa Aesar. Formamidinium iodide (FAI, 99.5%), methylamine chloride (MACl), lead (II) iodide (PbI_2_, 99.999%), phenethylammonium iodide (PEAI), methylammonium bromide (MABr), cesium iodide (CsI, 99.999%), 4‐tertbutylpyridine (tBP), Li‐bis‐(trifluoromethanesulfonyl)imide (Li‐TFSI), PCBM (99.5%) and Spiro‐OMeTAD (99.5%) were all brought from Xi'an Yuri Solar Co., Ltd. N, N‐dimethylformamide (DMF, 99.8%) and isopropanol (IPA, 99.5%) were purchased from Sigma–Aldrich. Trioctylphosphine oxide (TOPO, 98%), acetonitrile (ACN, 99.8%), Dimethyl sulfoxide (DMSO, 99.9%) and chlorobenzene (CB, 99.8%) were all purchased from Aladdin.

### Device Fabrication

The FTO substrates were sequentially ultrasonic cleaned with glass cleaner, deionized water, anhydrous ethanol, acetone and isopropyl alcohol for 20 min to remove impurities and organics. Then, they were subjected to UV‐ozone treatment for 20 min. The SnO_2_ colloid diluted with deionized water (1:5) was spin‐coated on the FTO substrates (3000 rpm for 30 s) and annealed at 150 °C for 30 min. Before spinning the perovskite precursor solution, the substrates were treated with UV‐ozone again for 15 min. After that, 599 mg of PbI_2_ dissolved in a mixed solvent DMF: DMSO (9:1; v/v) was spin‐coated on SnO_2_ substrate (2000 rpm for 30 s) and annealed at 70 °C for 1 min in a N_2_ glovebox. For comparison, different concentrations of CsI (2.5%, 5.0%, and 7.5% in mole) and TOPO (0.1%, 0.2%, and 0.3% in mole) were added to the PbI_2_ solution. Thereafter, a mixed organic ammonium solution FAI/MABr/MACl (120 mg: 12 mg: 12 mg dissolved in 2 mL IPA) was spin‐coated on PbI_2_ film (2000 rpm for 30 s), and then annealed in ambient air with a humidity of 20–30% at 150 °C for 20 min. After the formation of perovskite film, PEAI passivator (20 mm in IPA solution) was introduced on the surface of the film (5000 rpm for 30 s). Then, the Spiro‐OMeTAD solution was spin‐coated (3000 rpm for 30 s), which contains 72.25 mg of Spiro‐OMeTAD powder, 28.85 µL tBP and 17.5 µL Li‐TFSI solution (520 mg Li‐TFSI powder dissolved in 1 mL acetonitrile). At last, the gold electrode with a thickness of 100 nm was evaporated through a metal mask.

### Film and Device Characterizations

The XRD patterns were measured for analyzing the crystallinity and lattice strain of the films (Bruker D8 Advance, λ = 1.5406 Å). The UV–vis absorption spectra were obtained by the UV spectrometer (UV‐2600, Shimadzu). The surface morphologies of the films were observed using the SEM (model S‐4800, Hitachi) and AFM (model XE‐100E). Both XPS and UPS were measured by AXIS‐Ultra DLD (Kratos), with the binding energy corrected by the standard C1s peak (284.8 eV) for XPS measurement. The steady‐state PL and TRPL spectra of the films were measured by a PL spectrometer (Fluo Time 300, Pico Quant), with the excitation wavelength of 375 nm and frequency of 20 MHz. The Mott–Schottky measurement and EIS of PSCs were obtained (VersaSTAT 4, Princeton Applied Research). The illuminated *J–V* curves of the device were measured by a source meter (2400, Keithley) and a solar simulator (94022A, Newport) under standard condition (AM 1.5G, 100 mW cm^−2^, 25 °C). The *J–V* measurements were conducted by forward and reverse scans, with a scanning rate of 300 mV s^−1^. The effective area was 0.1 cm^2^ defined by a non‐reflective mask. The EQE spectra were obtained by the EQE measurement system (QEX10, PV Measurements).

## Conflict of Interest

The authors declare no conflict of interest.

## Supporting information

Supporting Information

Supporting Information

Supporting Information

## Data Availability

The data that support the findings of this study are available from the corresponding author upon reasonable request.
