# Supplementary material for: Strain Regulation and Defect Passivation of FA‐Based Perovskite Materials for Highly Efficient Solar Cells
Source: Adv Sci (Weinh). 2023 Dec 8;11(7):2305582. doi: 10.1002/advs.202305582 (PMC10870053; doi:10.1002/advs.202305582)
Supplement: Supplementary file 1 — Supporting Information [file ADVS-11-2305582-s001.pdf]

ADVANCED SCIENCE
Open Access

Supporting Information

for *Adv. Sci.*, DOI 10.1002/advs.202305582

Strain Regulation and Defect Passivation of FA-Based Perovskite Materials for Highly Efficient Solar Cells

Linfeng Zhang, Guohui Luo, Weihao Zhang, Yuxin Yao, Penghui Ren, Xiuhong Geng, Yi Zhang, Xiaoping Wu, Lingbo Xu, Ping Lin, Xuegong Yu, Peng Wang and Can Cui**

Supporting Information

Strain Regulation and Defect Passivation of FA-based Perovskite Materials for Highly Efficient Solar Cells

Linfeng Zhang[1], Guohui Luo[1], Weihao Zhang[1], Yuxin Yao[2], Penghui Ren[1], Xiuhong Geng[1], Yi Zhang[1], Xiaoping Wu[1], Lingbo Xu[1], Ping Lin[1], Xuegong Yu[2], Peng Wang[1*], Can Cui[1*]

L. Zhang, G. Luo, W. Zhang, P. Ren, X. Geng, Y. Zhang, X. Wu, L. Xu, P. Lin, P. Wang, C. Cui
Key Laboratory of Optical Field Manipulation of Zhejiang Province, Department of Physics, Zhejiang Sci-Tech University, Hangzhou, 310018, China
Y. Yao, X. Yu
State Key Laboratory of Silicon and Advanced Semiconductor Materials & School of Materials Science and Engineering, Zhejiang University, Hangzhou 310027, China

E-mail: pengwang@zstu.edu.cn, cancui@zstu.edu.cn

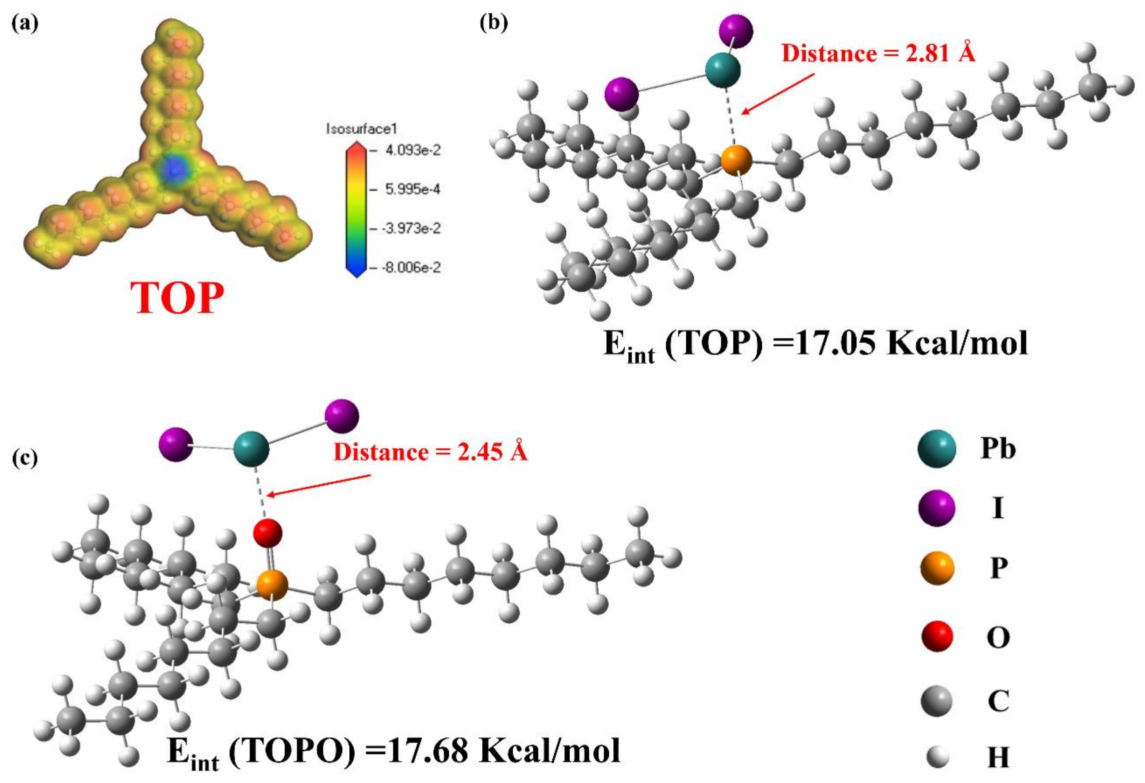

Figure S1. (a) ESP map of trioctylphosphine (TOP). The DFT calculation of interaction energies between (b) TOP or (c) TOPO and PbI_2.

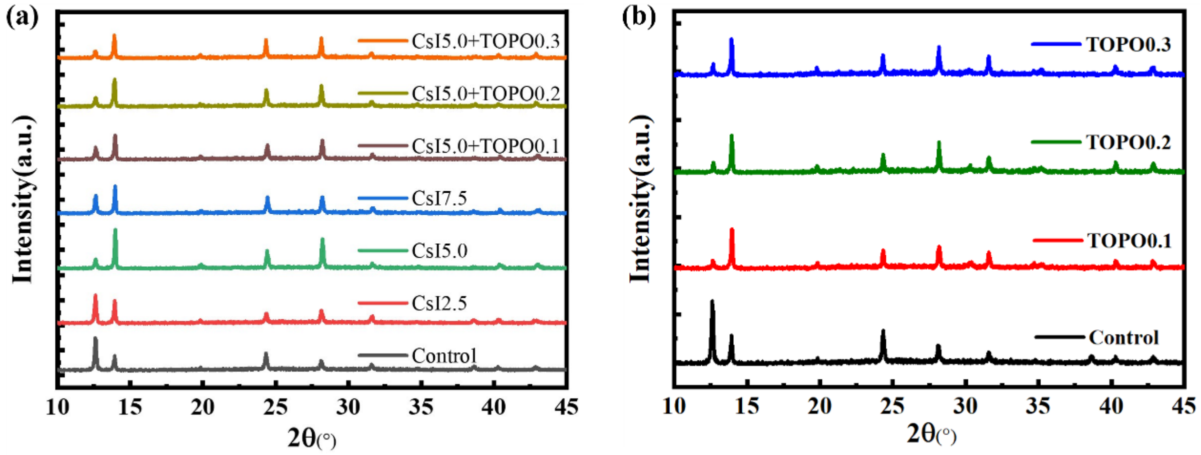

Figure S2. XRD patterns of the perovskite films: (a) the control, added with CsI, added with CsI and TOPO; (b) added with TOPO.

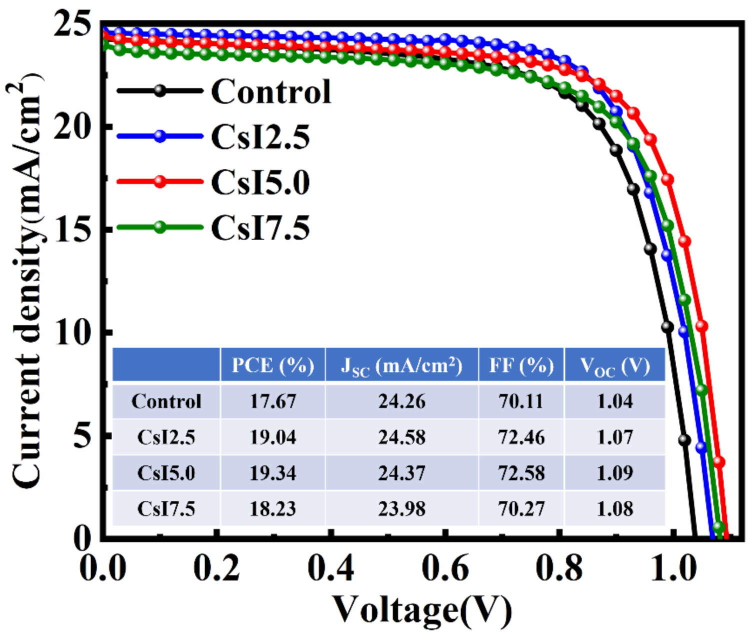

Figure S3. Illuminated J-V curves of the control and CsI added PSC devices.

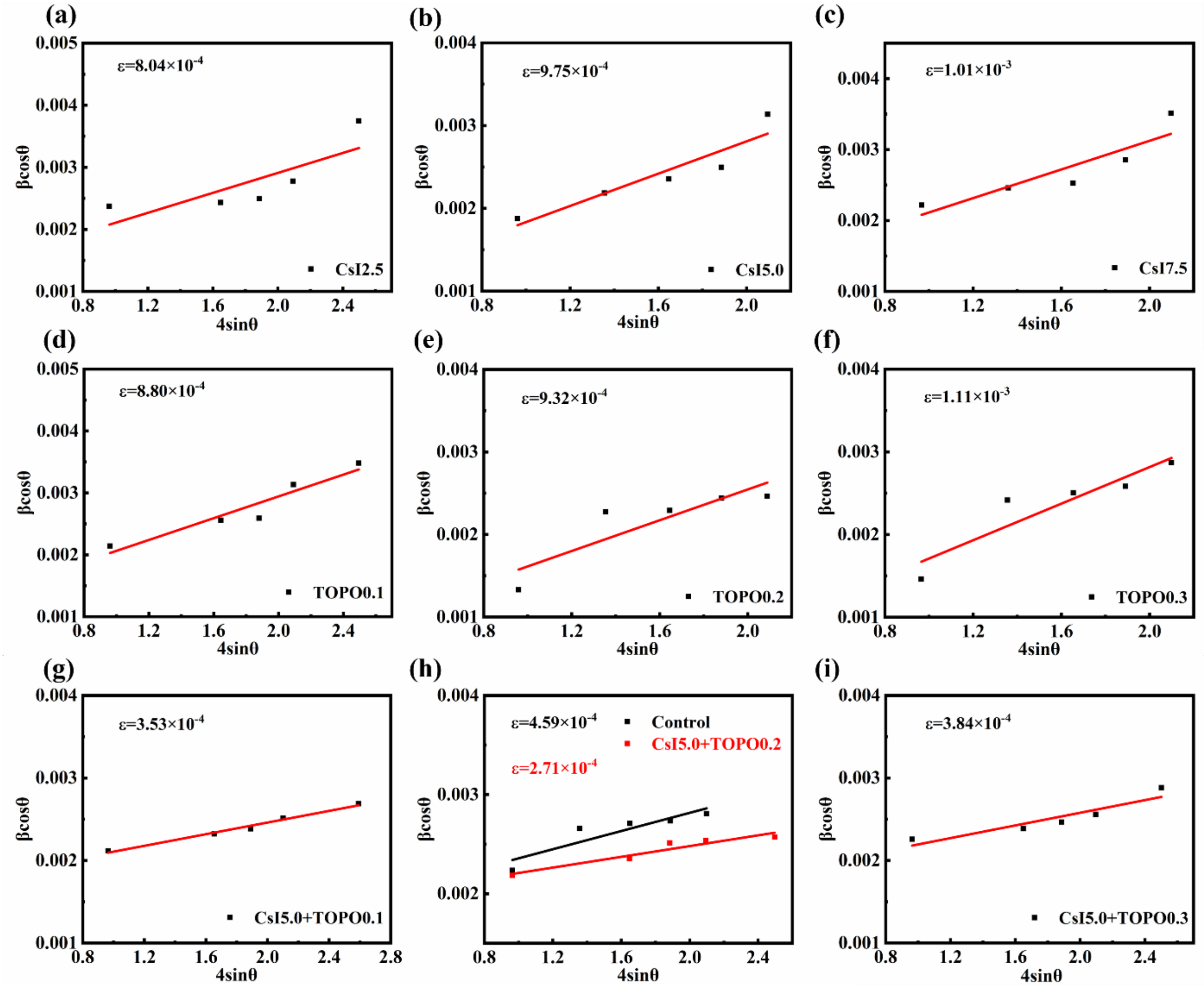

Figure S4. W-H plots of perovskite films: (a-c) added with CsI, (d-e) added with TOPO, (g-i) the control, added with CsI5.0 and TOPO.

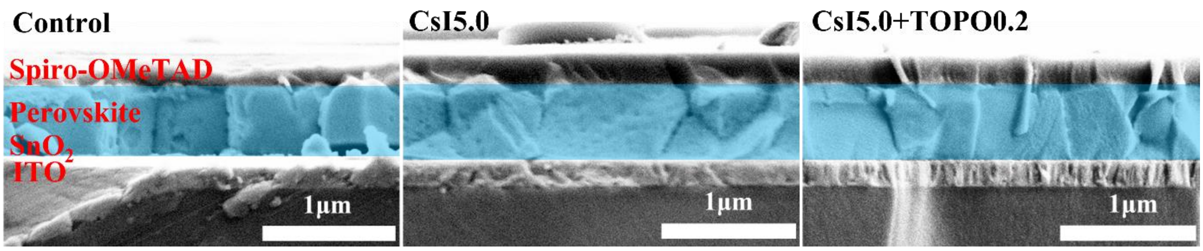

Figure S5. Cross-sectional SEM of the control, CsI5.0 and CsI5.0+TOPO0.2 films.

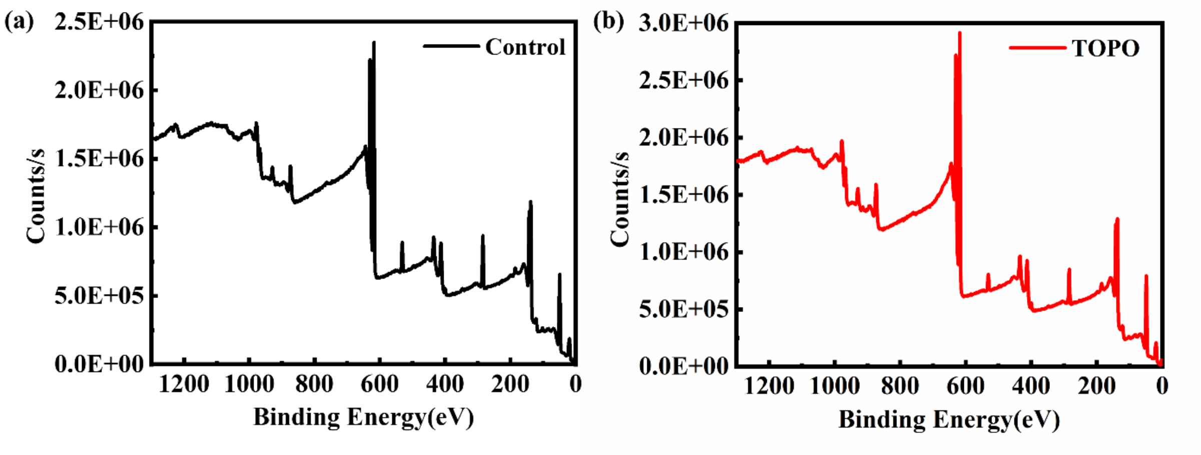

Figure S6. XPS full spectra of the perovskite films without (**a**) and with (**b**) the TOPO molecule.

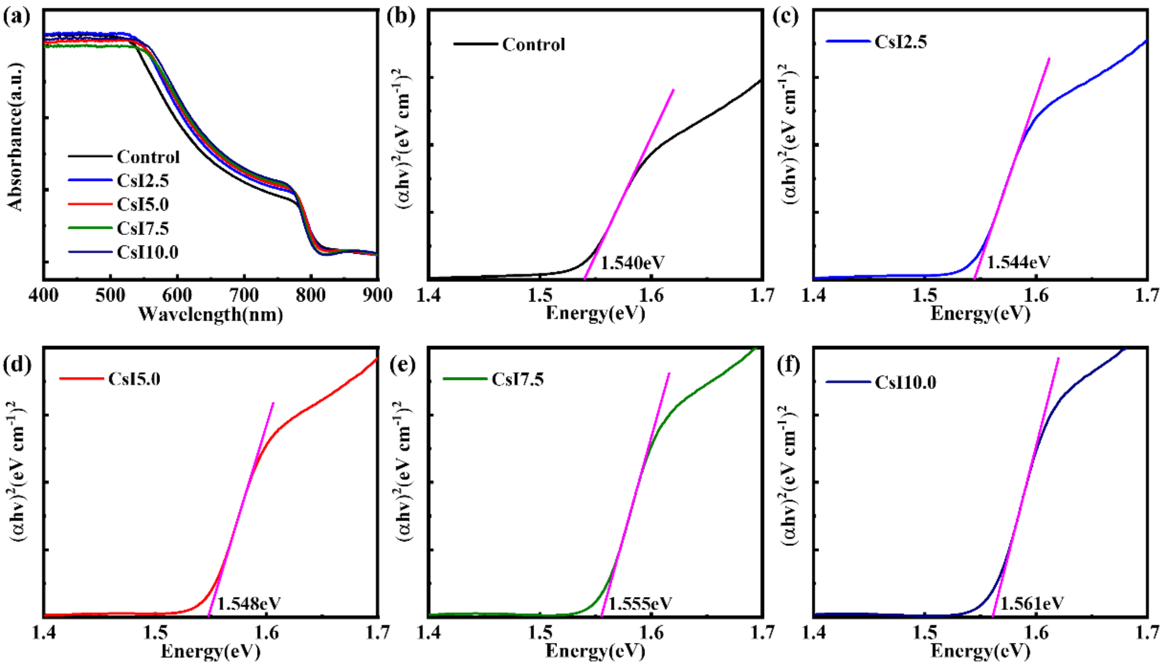

Figure S7. (a) UV-vis absorption spectra and (b-f) calculated bandgaps of the control and CsI doped films with different concentrations.

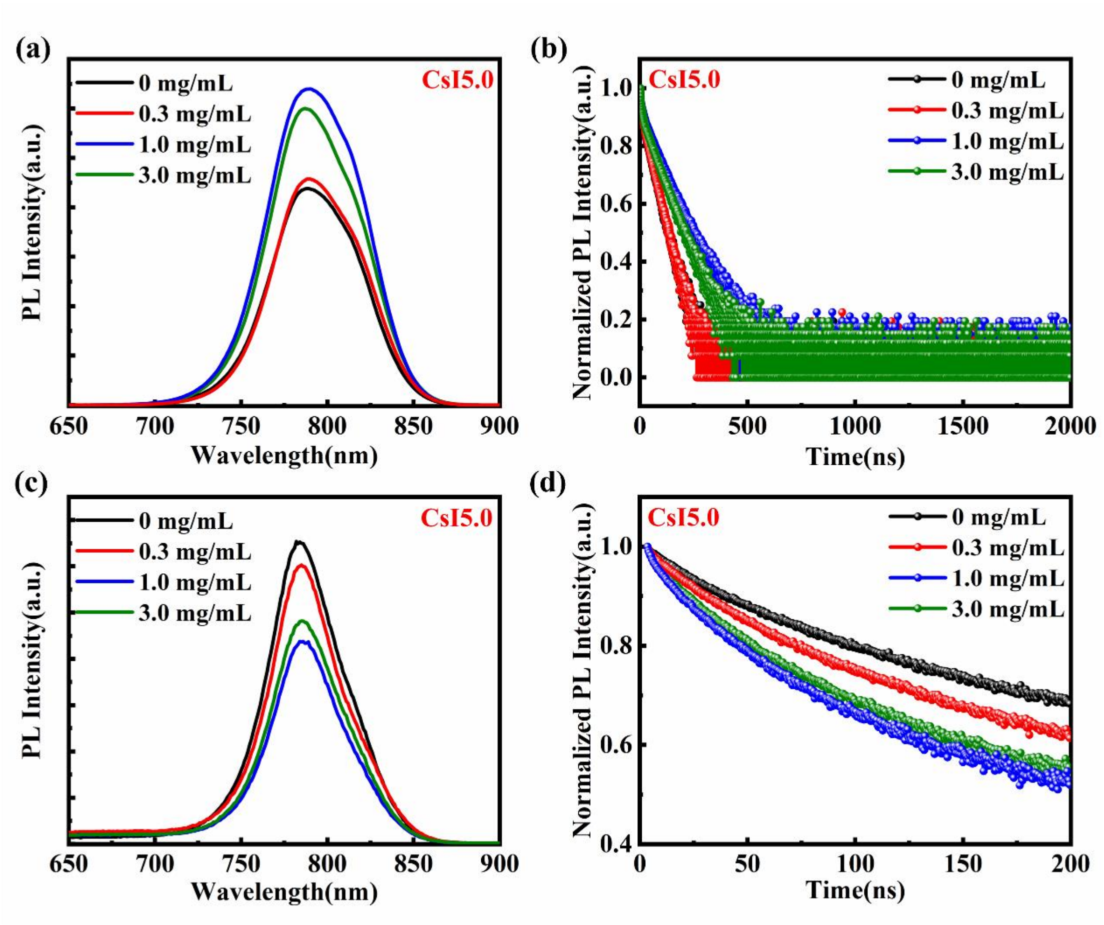

Figure S8. (a) PL and (b) TRPL spectra of CsI5.0 perovskite films deposited on glass, (c) PL and (d) TRPL spectra with the measured film structure of glass/perovskite/TOPO/spiro-OMeTAD for the introduction of TOPO at the surface with different concentrations.

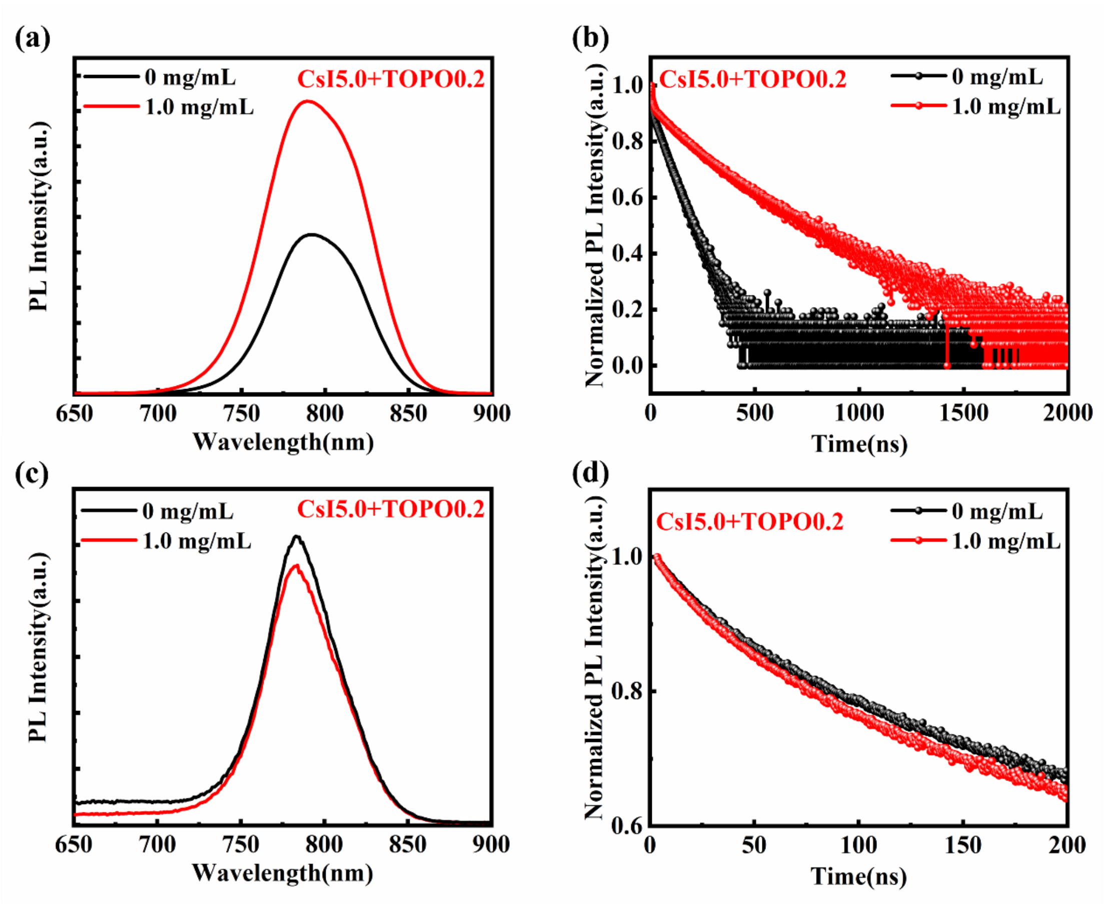

Figure S9. (a) PL and (b) TRPL spectra of target (CsI5.0+TOPO0.2) perovskite films deposited on glass, (c) PL and (d) TRPL spectra with the measured film structure of glass/perovskite/TOPO/spiro-OMeTAD for the introduction of TOPO at the surface with different concentrations.

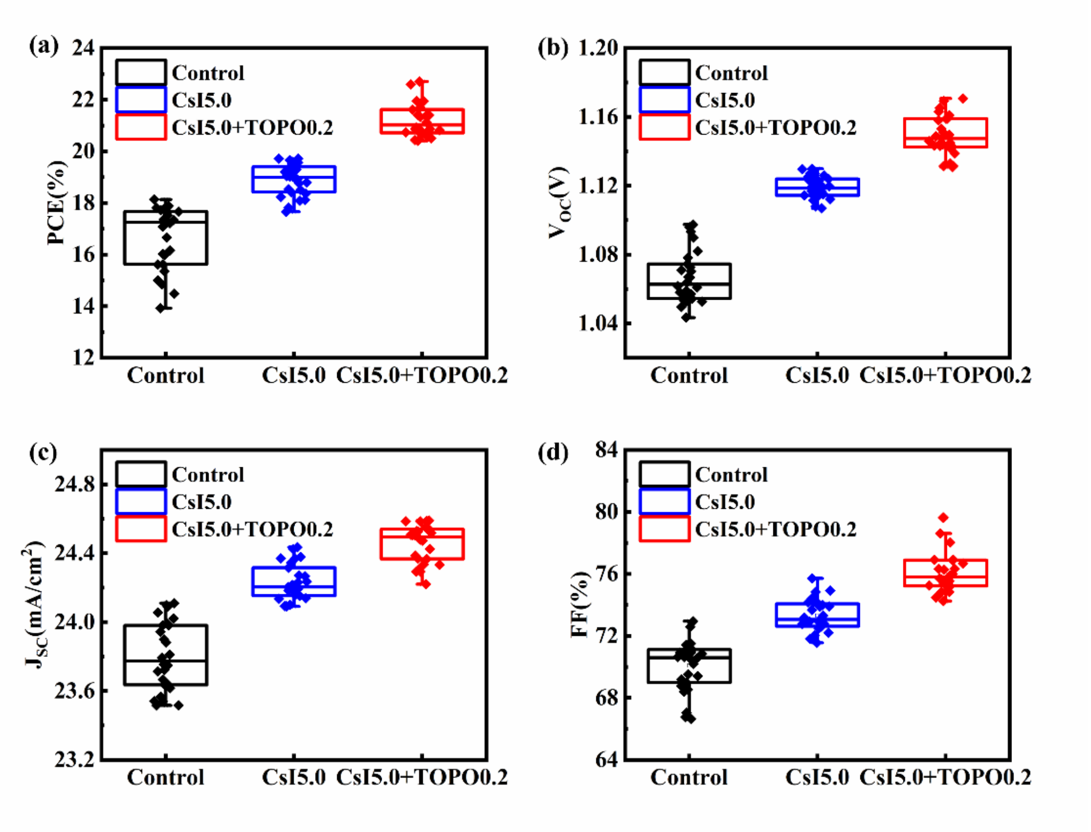

Figure S10. The statistical distributions of solar cell devices: (a) PCE, (b) V_{OC}, (c) J_{SC}, and (d) FF.

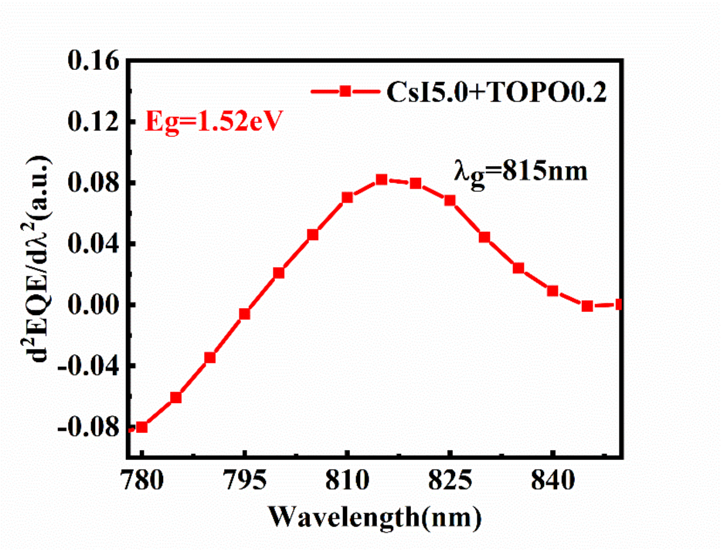

Figure S11. The fitted bandgap of the target FA-based perovskite from the second derivative of EQE with respect to wavelength λ.

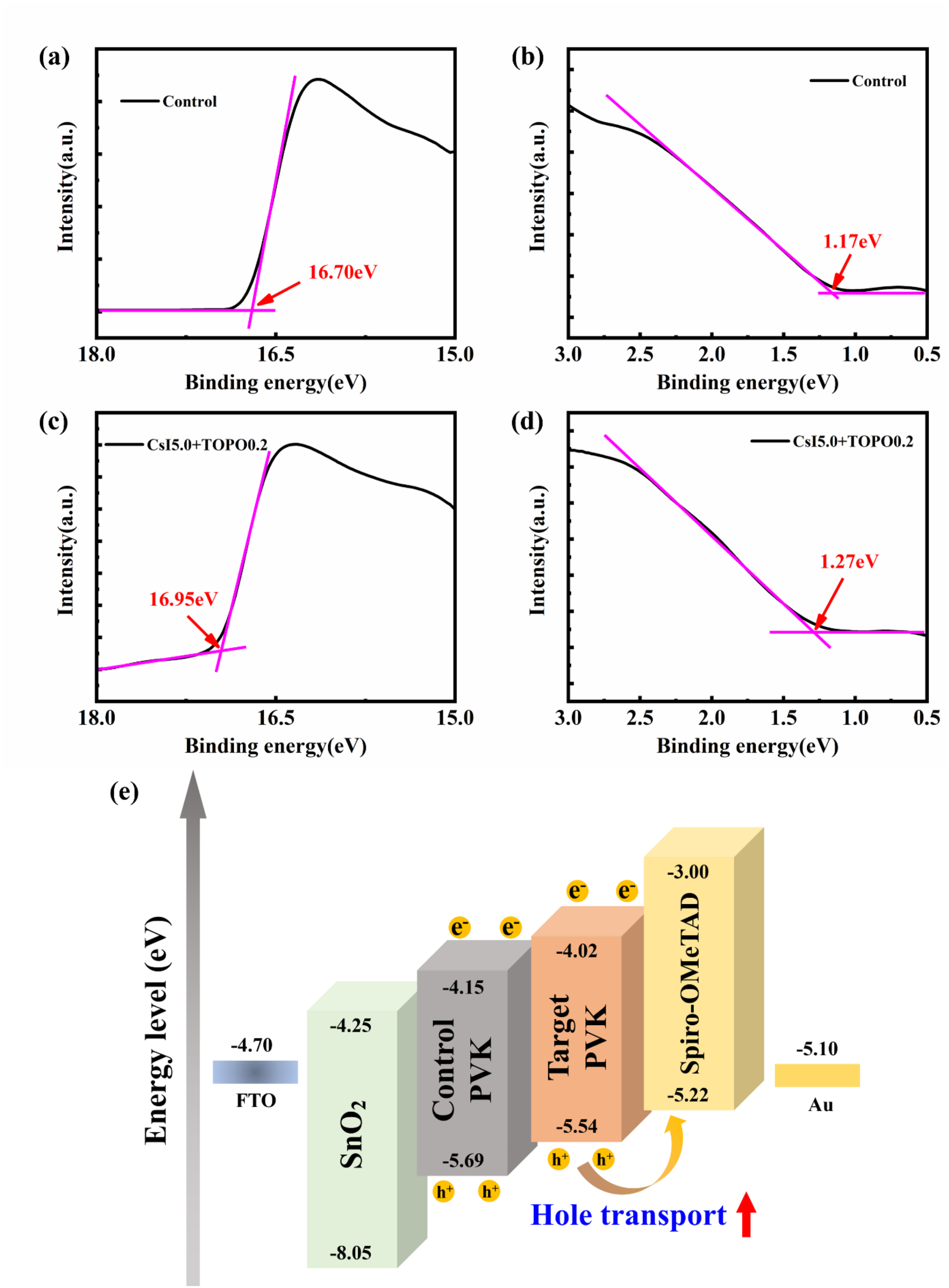

Figure S12. UPS analysis results for work function and valence band of the control (a-b) and target (CsI5.0+TOPO0.2) (c-d) perovskite. (e) Energy band diagram of the control and target device.

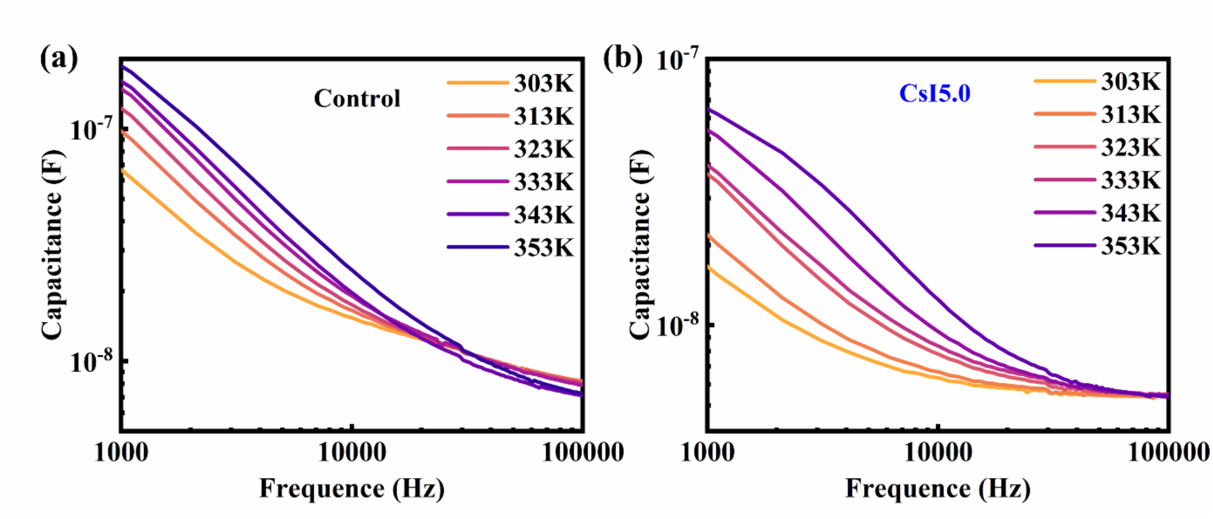

Figure S13. Capacitance-frequency curves at different temperatures of the control (**a**) and (**b**) CsI5.0 PSC devices.

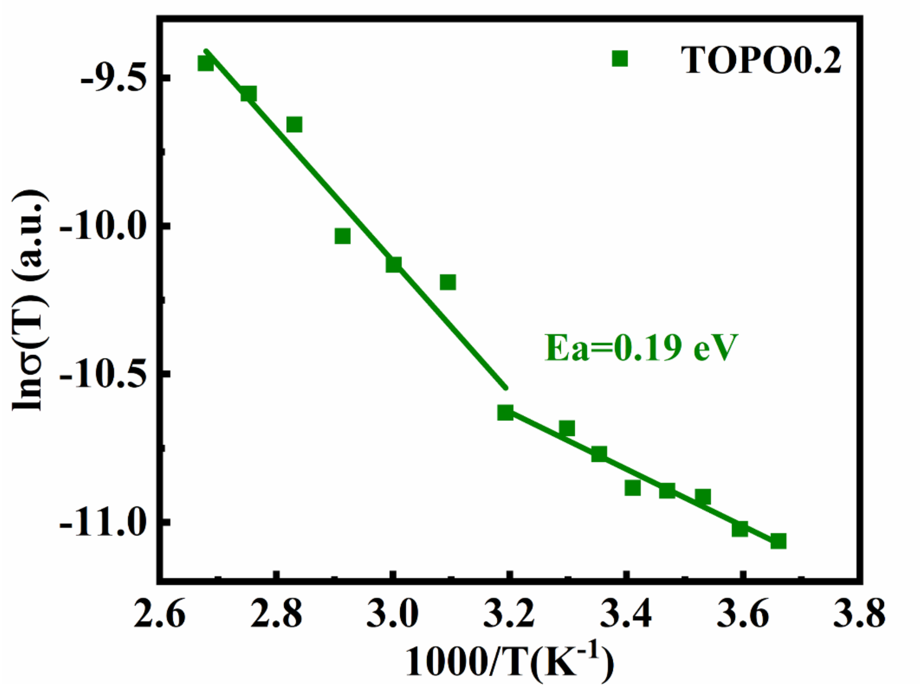

Figure S14. Temperature-dependent conductivity σ(T) of the TOPO0.2 device.

Table S1. The values of FWHM and **ε** calculated by XRD patterns of perovskite films for the control, added with CsI, TOPO or CsI5.0+TOPO.

	FWHM (°)	ε	Kλ/D
Control	0.164	4.59×10^{-4}	0.0019
CsI2.5	0.151	8.04×10^{-4}	0.0013
CsI5.0	0.144	9.75×10^{-4}	0.0009
CsI7.5	0.132	1.01×10^{-3}	0.0011
TOPO0.1	0.139	8.80×10^{-4}	0.0012
TOPO0.2	0.148	9.32×10^{-4}	0.0007
TOPO0.3	0.153	1.11×10^{-3}	0.0006
CsI5.0+TOPO0.1	0.132	3.53×10^{-4}	0.0018
CsI5.0+TOPO0.2	0.125	2.71×10^{-4}	0.0019
CsI5.0+TOPO0.3	0.131	3.84×10^{-4}	0.0018

Table S2. Fitted fast and slow decay components for the TRPL measurements of perovskite films for the control, added with CsI, TOPO or CsI5.0+TOPO deposited on glass substrates.

	A1	τ_1(ns)	A2	τ_2(ns)	τ_{avg}(ns)
Control	0.06	3.62	1.28	873.34	873.17
CsI2.5	0.24	8.72	0.71	1135.47	1132.55
CsI5.0	0.27	6.47	0.81	3095.26	3093.11
CsI7.5	0.27	6.66	0.70	2620.81	2618.25
TOPO0.1	0.07	16.15	1.30	983.54	982.69
TOPO0.2	0.03	109.84	0.85	2111.84	2108.17
TOPO0.3	0.04	18.72	0.97	1281.33	1280.57
CsI5.0+TOPO0.2	0.27	6.82	0.70	3216.95	3214.33

Table S3. Fitted fast and slow decay components for the TRPL measurements of perovskite films of the CsI5.0 and CsI5.0+TOPO0.2 modified with different TOPO concentrations deposited on glass substrates.

	A1	τ_1(ns)	A2	τ_2(ns)	τ_{avg}(ns)
CsI5.0-0 mg/mL	1.61	3.96	2.78	415.01	412.75
CsI5.0-0.3 mg/L	0.09	6.51	1.12	498.95	498.43
CsI5.0-1.0 mg/L	2.00	5.56	1.26	942.01	933.32
CsI5.0-3.0 mg/L	1.13	3.90	2.17	648.18	646.17
CsI5.0+TOPO0.2-0 mg/mL	2.01	5.13	0.12	870.68	792.93
CsI5.0+TOPO0.2-1.0 mg/L	0.01	60.94	1.15	1895.53	1895.02

Table S4. Fitted fast and slow decay components for the TRPL measurements of the films with the structure of glass/perovskite/TOPO/spiro-OMeTAD.

	A1	τ_1(ns)	A2	τ_2(ns)	τ_{avg}(ns)
CsI5.0-0 mg/mL	0.01	19.17	0.44	220.75	220.35
CsI5.0-0.3 mg/L	0.03	31.23	0.51	211.18	209.63
CsI5.0-1.0 mg/L	0.01	9.02	0.52	138.64	138.48
CsI5.0-3.0 mg/L	0.02	9.16	0.52	125.92	125.59
CsI5.0+TOPO0.2-0 mg/mL	0.03	27.60	0.45	247.07	245.45
CsI5.0+TOPO0.2-1.0 mg/mL	0.03	22.35	0.43	182.83	181.47
